# Protection against Dextran Sulfate Sodium-Induced Ulcerative Colitis in Mice by Neferine, A Natural Product from *Nelumbo nucifera Gaertn*

**DOI:** 10.22074/cellj.2021.6918

**Published:** 2020-04-22

**Authors:** Xiangjing Min, Yanling Guo, Yishan Zhou, Xiuping Chen

**Affiliations:** 1 Key Lab for Pharmacology of Ministry of Education, Department of Pharmacology, Zunyi Medical University, Zunyi, China; 2 State Key Laboratory of Quality Research in Chinese Medicine, Institute of Chinese Medical Sciences, University of Macau, Macao, China

**Keywords:** Dextran Sulfate Sodium, Inflammation, Neferine, Ulcerative Colitis

## Abstract

**Objective:**

Ulcerative colitis (UC) is a long-lasting inflammatory disease of the colon. Epidemiological studies showed that the prevalence and incidence of UC are increasing worldwide in recent years. Neferine is a natural alkaloid isolated from Nelumbo nucifera Gaertn that exerts a variety of biological activities. This study was designed to evaluate the protective effect of neferine on dextran sulfate sodium (DSS)-induced experimental UC in mice.

**Materials and Methods:**

In this experimental study, 4% DSS was used to induce a mice model of UC. Neferine (5 and 10 mg/kg) was administered by intraperitoneal injection (ip). Clinical symptoms and disease activity index (DAI) scores were recorded and calculated. Pathological changes of colon tissues were detected by Hematoxylin and Eosin (H&E) staining. The levels of inflammatory mediators were detected by ELISA kits. Western blotting and immunohistochemical analysis were used for the evaluation of protein expressions.

**Results:**

Neferine treatment significantly alleviated DSS-induced UC by inhibiting weight loss, decreasing DAI scores, and alleviating the pathological changes in colon tissues. Furthermore, neferine significantly decreased serum levels of pro-inflammatory cytokines including tumor necrosis factor-alpha (TNF-α), interleukin-1 beta (IL-1β), and IL-6 and increased serum levels of anti-inflammatory cytokine IL-10. The increased myeloperoxidase (MPO) activity and nitric oxide (NO) in colon tissues were also inhibited. In addition, neferine significantly down-regulated inducible NO synthase (iNOS), cyclooxygenase-2 (COX-2), and intercellular cell adhesion molecule-1 (ICAM-1) expression in colon tissues.

**Conclusion:**

These results provided evidence that neferine could protect against DSS-induced UC symptoms in an experimental mice model. This effect might be mediated through inhibition of inflammation.

## Introduction

Ulcerative colitis (UC), a form of inflammatory bowel disease (IBD), is a chronic inflammatory disease (1). UC was first described by Wilks and Moxon in 1875 and officially named by the International Organization Committee of the World Health Organization (WHO) Medical Science Organization in 1973. Clinically, UC is characterized by diarrhea, abdominal pain, mucus pus, bloody stools, and acute diarrhea (2). The lesion site of UC is mostly confined to colon mucosa, including colon mucosa and submucosa, which may involve rectum and distal colon, and spread to the proximal colon and subsequently spread throughout the entire colon, showing a continuous and diffuse distribution (3).

The risk factors and pathogenesis of UC are still unclear (4). It was pointed out that the pathogenesis of the disease is complicated and is related to multiple factors, such as genetic factors, immune factors, infection factors, and inflammatory mediators (5). Epidemiological studies showed that the prevalence and incidence of UC are increasing worldwide in recent years (6). UC has the characteristic of recurrence and there is still a lack of effective and safe therapeutic drugs for treatment of the disease (7). Therefore, finding safe and effective drugs against this serious disease is of particular interest to pharmaceutical companies and researchers.

**Fig.1:**
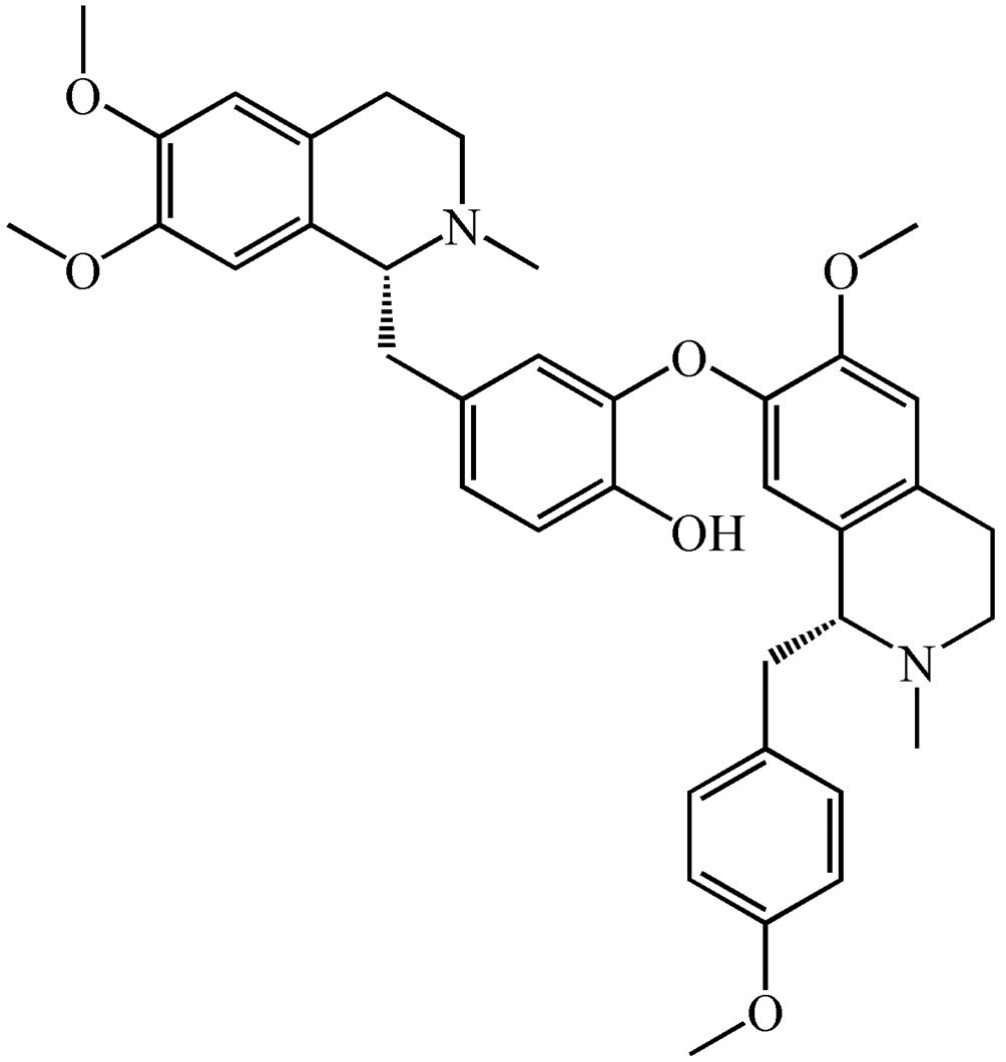
The chemical structure of neferine, a natural alkaloid (10).

Traditional Chinese medicines (TCM) has a long history of clinical application for the treatment of various diseases. Especially, documented data demonstrated that TCM and TCM-derived natural products showed protective effects on UC (8, 9). Neferine, a dibenzyl isoquinoline alkaloid (Fig.1), is extracted from the mature seed embryos of *Nelumbo nucifera* Gaertn (Lotus) (10). Numerous studies revealed that neferine possesses various biological activities, such as anti-angiogenesis (11), anti-oxidant (12), anti-cancer (13), anti-diabetic (14, 15), anti-thrombotic, anti-arrhythmic (14), antiinflammatory (16) and anti-HIV properties (14, 17). We recently reported that neferine significantly inhibited LPS-induced inflammation in Raw264.7 cells and oral administration of neferine improved DSS-induced inflammation in mice (16). However, the underlying mechanisms remain unclear. Here, neferine protective effects and the underlying mechanism(s) were further investigated following intraperitoneal injection (ip) of neferine in a DSS-induced mice model.

## Materials and Methods

### Reagents

In this experimental study, Neferine (>95%) was provided by Chenguang Herb purify Co., Ltd. (Chengdu, China). Dextran sulfate sodium (DSS, MW 36,000-50,000 Da) was purchased from MP Biomedicals (California, USA). LOX-1, iNOS, COX-2 antibodies were obtained from Abcam (Cambridge Science Park, UK). Antibodies for ICAM-1, VCAM-1, NOSTRIN, β-actin, and GAPDH were purchased from Proteintech (Wuhan, China). ELISA kits for myeloperoxidase (MPO) and NO were purchased fromNanjing Jiancheng Bioengineering Institute Co., Ltd. (Nanjing, China). ELISA kits for tumor necrosis factor-alpha (TNF-α), interleukin-1beta (IL-1β), IL-10, and IL-6 were bought from Shanghai Jianglai Biological Technology Co., Ltd. (Shanghai, China).

### Animals and experimental design

C57BL/6J male mice (body weight 22-24 g) purchased from the Chongqing Tengxin Biotechnology Co., Ltd. (Chongqing, China), were maintained in specific pathogenfree (SPF) environment (temperature 24-25˚C, humidity 50- 55%，12 hours/12 hours light/dark cycle). Mice were fed with a normal laboratory diet and water ad libitum. They were acclimated to the laboratory environmental conditions for two weeks before the experiments. All protocols were performed upon approval by the Ethics Committee of Zunyi Medical University and were strictly performed in accordance with the "Guide for the Care and Use of Laboratory Animals" (National Research Council, 2011, 81774200).

**Fig.2:**
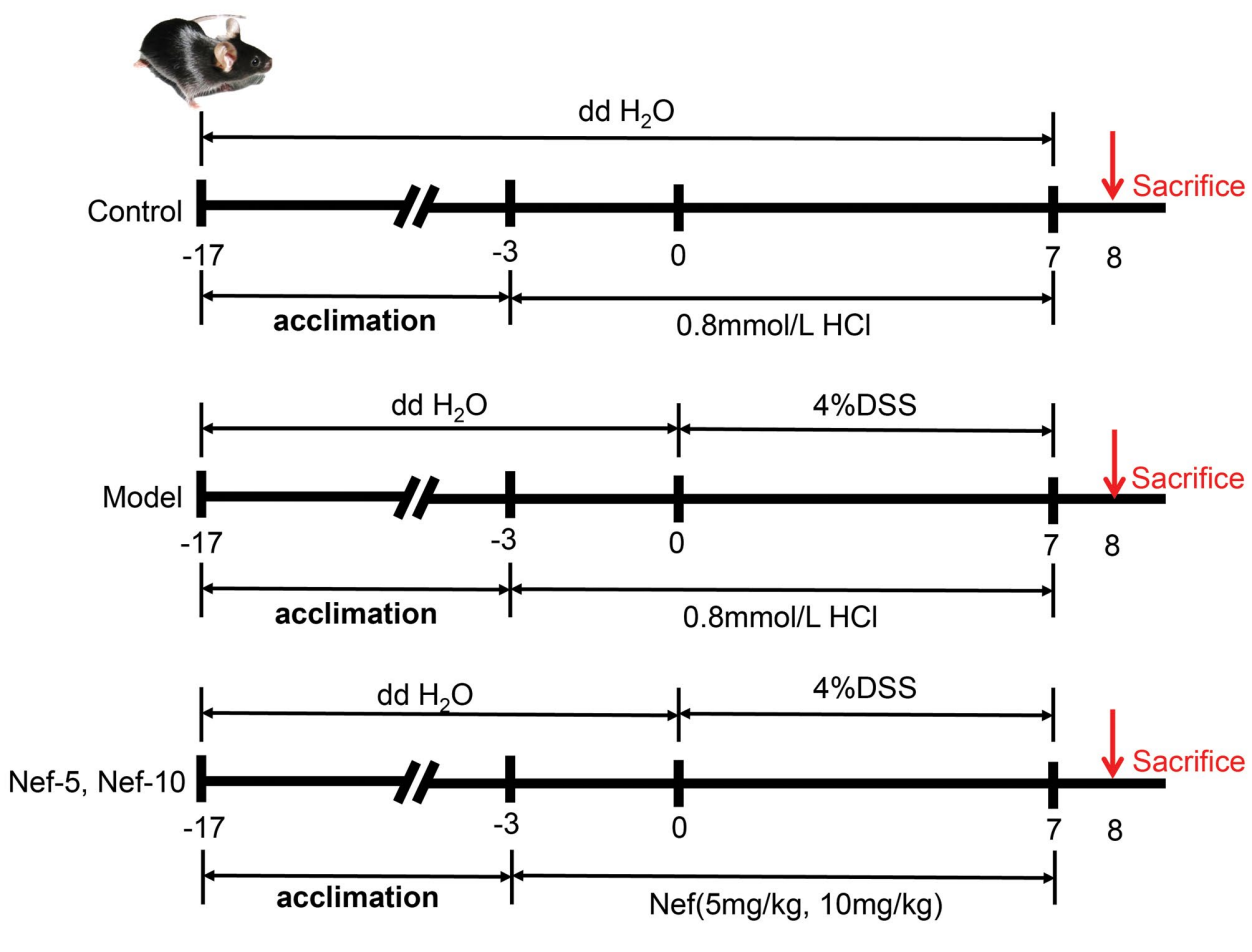
The schematic experimental design of the present study. After 2 weeks of adaptive feeding, mice were randomly divided into groups according to body weights. The experimental period was a total of ten days, including three days of neferine pretreatment by ip followed by co-treatment of 4% dextran sulfate sodium (DSS) and neferine for another 7 consecutive days.

Induction of UC was conducted as shown in our previous report with minor revisions. Thirty-four mice were randomly divided into four groups: the control group (n=8), the model group (n=10), the low dose (5 mg/kg) group (n=8) and the high dose (10 mg/kg) group (n=8). Mice in the control group received water only while mice in other groups were administered with 4% DSS in drinking water for 7 consecutive days. Mice in neferine-treated groups received neferine ip daily for 10 days, started 3 days before and continued for 7 days after administration of 4% DSS. Neferine was dissolved in 0.8 mM HCl. The colons and serum were collected after mice were sacrificed. The experimental design is summarized in Figure 2.

### Disease activity index

The progression of UC was evaluated daily by calculating the scores during the course of treatments as previously described (18), according to the percentage change in body weight, the severity of fecal bleeding and the occurrence of diarrhea.

### Hematoxylin and eosin staining

Hematoxylin and eosin (H&E) staining was performed as previously shown (19, 20) with minor revisions. Briefly, colons tissues were immediately fixed in 10% paraformaldehyde overnight and then, embedded in paraffin and sectioned at 4 ىm thickness. Slides were then stained with H&E and examined using a microscope for histopathological alterations.

### Immunohistochemical assay

The expression of ICAM-1 was investigated using immunohistochemical analysis based on a previous report (16) with minor revisions. Briefly, the colon tissue sections were dried at 60˚C for 45 minutes. Then, the fixed sections were deparaffinized and placed in medium-high heat oven for 18 minutes for antigen unmasking. After inhibition of endogenous peroxidase activity by 3% H_2_O_2_ solution, non-specific antigens were inhibited by 5% universal blocker for 30 minutes. The slides were incubated with primary antibodies for ICAM-1 (1:200) overnight at 4˚C and then, washed with PBS twice. The slides were incubated for 30 minutes with biotinylated universal link second antibody, stained with 3,3’-Diaminobenzidine (DAB), counterstained with hematoxylin, dehydrated and mounted. Finally, sections were observed under a microscope and images were obtained.

### Determination of myeloperoxidase activity and nitric oxide level in colon tissues

MPO activity and NO levels in colon tissues were determined using commercial kits according to the manufacturer’s protocol.

### Determination of cytokines

Cytokines (TNF-α, IL-1β, IL-10, and IL-6) levels in serum were determined by commercial ELISA kits following the manufacturer’s recommendations.

### Western blotting

Colon tissues from different groups were homogenized on ice to extract proteins using protein lysis buffer (containing radio immunoprecipitation assay (RIPA buffer), phenylmethanesulfonyl fluoride (PMSF, 0.1 M), and protease inhibitors). Protein concentrations were determined using BCA protein kit. Equal samples from each group were isolated by 8% sodium dodecyl sulphate-polyacrylamide gel electrophoresis (SDS-PAGE) and then, transferred to the polyvinylidene difluoride (PVDF) membrane. After blocking the PVDF membrane with 5% nonfat milk for 2 hours, primary antibodies (β-actin (1:2000), GAPDH (1:2000), iNOS (1:500), COX-2 (1:500), ICAM-1 (1:1000), VCAM- 1 (1:1000), LOX-1 (1:1000) and ROSTRIN (1:1000)) were added. Subsequently, after incubation with the secondary antibody for 1 hour, chemiluminescence signals were detected by ChemiDoc™ Imager image scanner (Bio-Rad Biotech, CA, USA).

### Statistical analysis

Results are expressed as means ± standard deviation (SD). Statistical analysis was performed by one-way analysis of variance (ANOVA) by the SPSS 18.0 software software (IBM SPSS, USA). Differences with P<0.05 were considered statistically significant.

## Results

### Neferine alleviated the symptoms of dextran sulfate sodium -induced colitis

**Fig.3:**
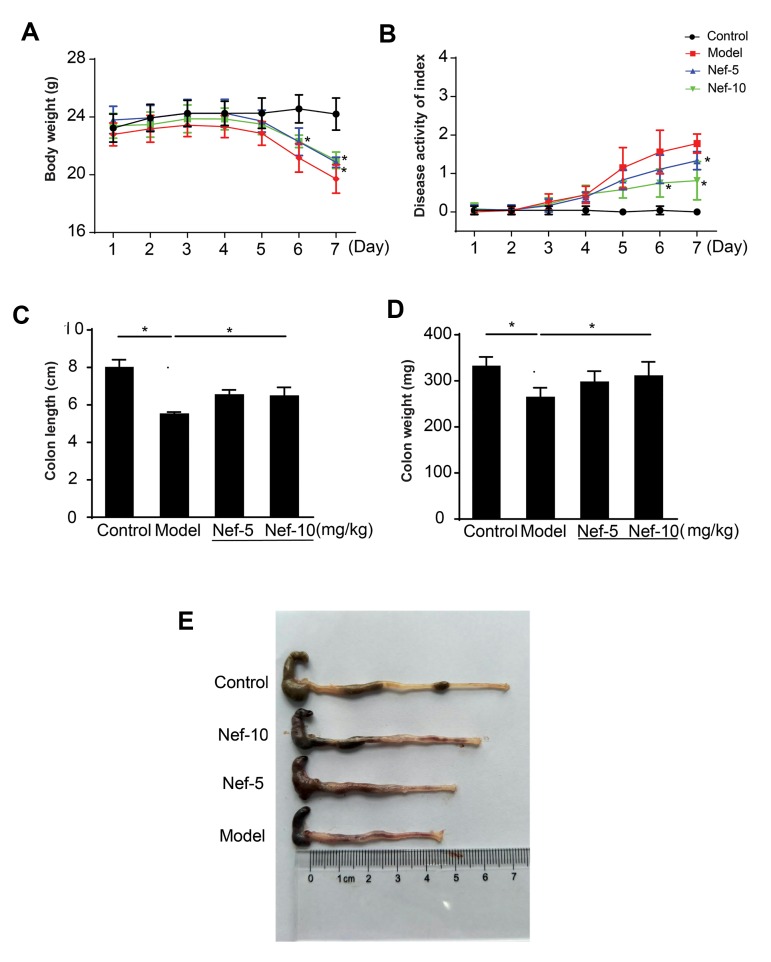
Neferine improved UC induced by DSS in mice. Three days after prophylactic intraperitoneal injection of neferine, mice were challenged with 4% DSS for 7 days of ulcerative colitis modeling. **A.** The average body weights, rectal bleeding and diarrhea were recorded daily. **B.** DAI scores were calculated. **C.** Mean colon lengths. **D.** Average colon weights. **E.** Representative pictures of colon. *; P<0.05 control versus model, model versus neferine, UC; Ulcerative colitis, DSS; Dextran sulfate sodium, and DAI; Disease activity index.

Compared with the control group, the body weights of the model group significantly decreased from day 5 to 7. Neferine pretreatment partially inhibited DSS-induced body weights loss (Fig.3A). On the third day after the DSS treatment, diarrhea and bloody stools were observed in some mice. Compared with the control group, DAI scores in model group were significantly increased from day 5 to 7, which was partially reversed by neferine pretreatment (Fig.3B). Furthermore, compared with the control group, the colons from the model group were obviously contracted (Fig.3C, D) and the weights of the colons were also dramatically decreased. These alterations were significantly improved in neferine-administered groups.

### Neferine improved dextran sulfate sodium -induced pathological changes of colitis

**Fig.4:**
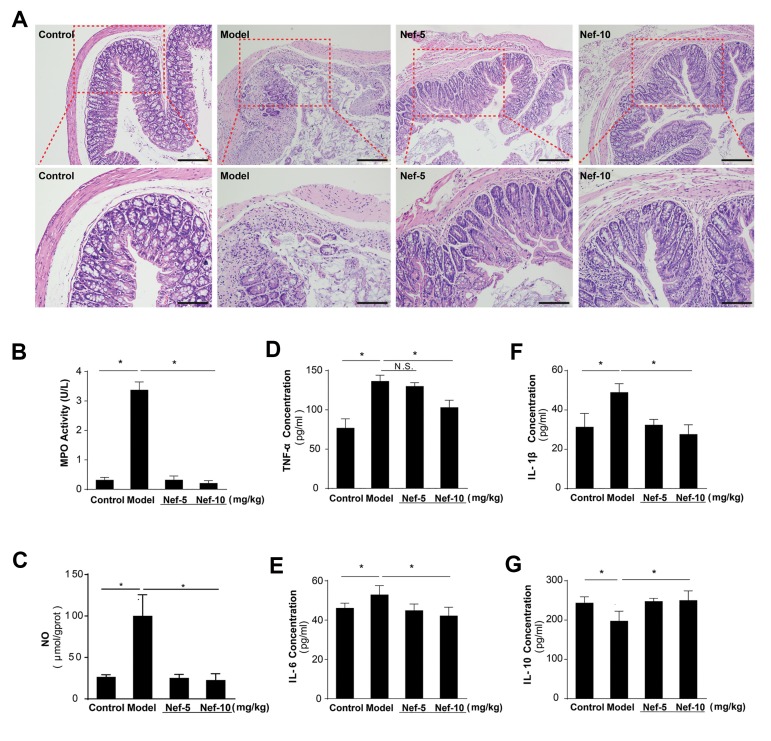
Neferine improved histological changes and restored inflammatory mediators in UC mice. **A.** Representative images of H&E staining of colon tissues are presented [the magnifications of the upper and lower panels are ×100 (scale: 100 μm) and ×200 (scale: 100 μm), respectively]. **B.** The MPO activity and **C.** NO content in colon tissues were determined. Serum levels of **D.** TNF-α, **E.** IL-1β, **F.** IL-6, and **G.** IL-10 were determined. *; P<0.05, control versus model, model versus neferine, UC; Ulcerative colitis, MPO; Myeloperoxidase, NO; Nitric oxide, TNF-α; Tumor necrosis factoralpha, and IL; Interleukin.

H&E staining showed that the physiological structures of colon tissue from the control group were integral and clear with arranged goblet cells in the mucosa. However, the colon structures in the model group were severely damaged with decreased number of goblet cells, mucosal ulcers, and increased infiltration of neutrophils. These pathological changes were partially improved by neferine administration, especially in the high dose group (Fig.4A).

### Neferine decreased myeloperoxidase activity and regulated inflammatory cytokines secretion

The MPO activities in the model group approximately increased 7 folds, which was almost completely inhibited by neferine at both doses (Fig.4B). Similar inhibitory effects were observed in terms of DSS-induced NO increase in colon tissues (Fig.4C). Furthermore, the serum levels of pro-inflammatory cytokines TNF-α, IL-1β, and IL-6 were significantly increased in the model group, which was inhibited by neferine treatment (Fig.4D-F). In addition, the serum levels of IL-10 in the model group were significantly decreased, which was partially reversed by neferine treatment (Fig.4G).

### Neferine inhibited COX-2 and iNOS expression

**Fig.5:**
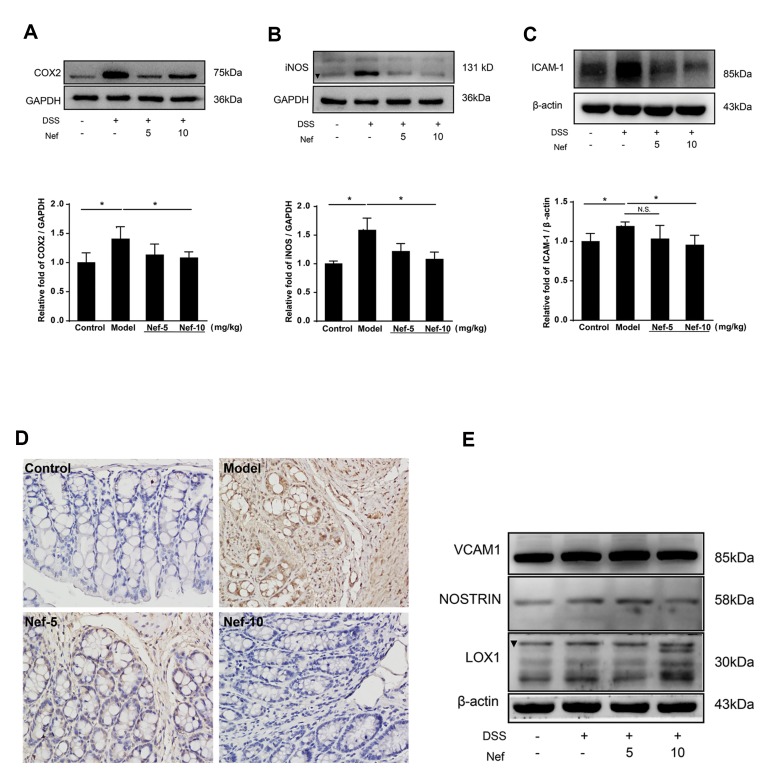
Effect of neferine on protein expression in colon tissues. The protein expression of **A.** COX-2, **B.** iNOS, **C.** ICAM-1 in the colon tissues was determined by Western blotting. **D.** The expression of ICAM-1 in colon tissues was detected by immunohistochemical analysis (×400, scale bar: 50 μm). **E.** The protein expression of VCAM-1, NOSTRIN, and LOX-1 was determined by Western blotting. *; P<0.05, control versus model, model versus neferine.

Western blotting results showed that the protein expression of COX-2 in colon tissues in the model group was significantly increased, which was significantly inhibited by neferine (Fig.5A). Similarly, the protein expression of iNOS in the model group was dramatically increased, which was significantly reversed by neferine (Fig.5B).

### Neferine restored dextran sulfate sodium -induced expression of ICAM-1 protein

The protein expression of ICAM-1 in colon tissues in the model group was significantly upregulated, which was completely inhibited by neferine at both doses (Fig.5C). In immunohistochemical analysis, weak brown staining was observed in the control colon tissues while in the model tissues, dramatically enhanced brown staining was observed. Furthermore, treatment with both doses of neferine significantly decreased the brown staining (Fig.5D). However, no difference in the expression levels of VCAM-1, NOSTRIN, and LOX-1 among the groups was found (Fig.5E). These results indicated that the expression of ICAM-1 in the model group was upregulated, but decreased by neferine.

## Discussion

UC is a digestive tract disease characterized by chronic inflammation and ulceration of colonic mucosa and submucosa. Drugs available for UC treatment in clinic are mainly salicylic acid, glucocorticoids, immunosuppressive agents and biological agents (21). Recent studies suggested that many medicinal plants and natural products might have therapeutic potentials for UC (22). Neferine is a natural alkaloid with various pharmacological effects (14) and our recent study showed that oral administration of neferine protects against DSS-induced UC; however, exact mechanism(s) remain unclear (16). In the present study, neferine protective effect on DSS-induced UC and the underlying mechanism were further explored. The main findings of this study were: i. Intraperitoneal injection of neferine significantly protects against DSS-induced UC in mice and ii. This protective effect was mediated via regulation of cytokines secretion and iNOS, COX-2 and ICAM-1 expression.

Many chemicals have been used for induction of experimental UC, such as 2,4,6-trinitro-benzene sulfonic acid (TNBS), oxazolone, DSS and acetic acid (23). Because DSS-induced UC model exhibits similar clinical symptoms and pathological features to those of human IBD, this model has been widely used in basic research (18, 24, 25). In this model of UC, two phases were observed. During the active period, mice had hair erect, weight loss, diarrhea, blood in the stool, and the occurrence of death. Histological changes include changes in mucin depletion, crypt structure, epithelial cell changes, and infiltration of inflammatory cells (25). Here, DSS-treated mice showed decreased body weight, increased DAI scores and decreased colon length and weight. H&E staining showed mucin depletion, epithelial degeneration and infiltration of inflammatory cells. These suggested that the UC model was successfully established. Similar to our previous report and other reports about UC (16, 26), neferine treatment could significantly improve these clinical manifestations and histological alterations. Thus, both oral administration and intraperitoneal injection of neferine showed protective effects in this model. In view of the compliance in clinical, oral administration might be a better choice.

DSS could penetrate the mucosal membrane in the intestine. Lysosomes containing DSS molecules could be found in macrophages in the lamina propria of colon mucosa, and infiltrated inflammatory cells. Under the stimulation by DSS, macrophages on the intestinal surface were activated to produce pro-inflammatory cytokines, including TNF-α, IL- 1β, IL-6, which participate in the development of UC. COX-2 and iNOS, two inducible enzymes, produced by macrophages, play important roles in inflammatory responses, including UC (27). Here, neferine treatment significantly inhibited the pro-inflammatory cytokines secretion. Especially, the levels of IL-10, a cytokine with potent anti-inflammatory properties (28), were restored by neferine. Thus, neferine could regulate the balance of pro- and anti-inflammatory cytokines. Neferine decreased NO levels while showed no effect on the colon expression of NOSTRIN, a protein modulating activity, trafficking, and targeting of eNOS. This suggested that increased levels NO were secreted by iNOS. MPO is secreted by neutrophils, and changes in its activity in the colon can indirectly reflect the level of neutrophil infiltration in the colon (29). It could be a useful disease activity biomarker for several diseases, including UC (30). Increased MPO activity was completely inhibited by neferine which suggested that the disease activity could be improved by neferine.

**Fig.6:**
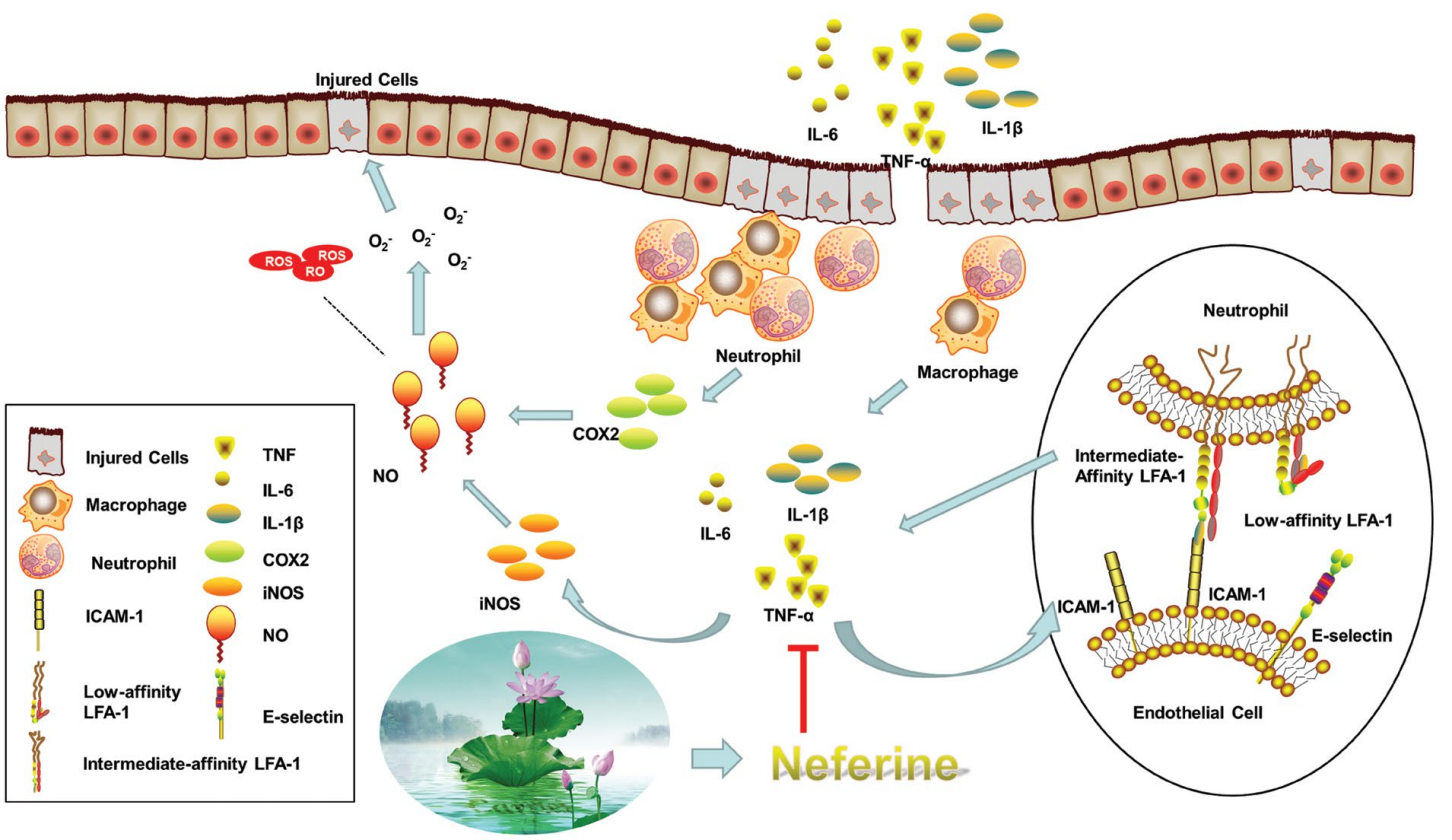
Schematic diagram for protective effect of neferine on UC. Neferine inhibited UC mainly by regulating the secretion of inflammatory cytokines (the proinflammatory cytokines TNF-α, IL-1β, and IL-6, and the anti-inflammatory cytokines, IL-10), and inhibiting the expression of COX-2, iNOS, and ICAM-1 proteins. UC; Ulcerative colitis, TNF-α; Tumor necrosis factor-alpha, and IL; Interleukin.

Integrins and adhesion molecules have been attractive targets for the treatment of IBD (31). Previous reports showed increased or unaltered expression of VCAM-1 in mucosa of IBD (32, 33). Here, no enhanced expression of VCAM-1 was observed in DSS-treated colon tissues. Furthermore, the expression of LOX-1, the scavenger receptor mainly found in endothelial cells (34, 35), was not altered by DSS. Thus, these results suggested that neither VCAM-1 nor LOX-1 was actively involved in DSS-induced colitis. ICAM-1, also known as CD54, is a single chain transmembrane glycoprotein. It is an adhesion factor closely related to colon mucosa cells (36). In normal colon tissues, the expression of ICAM- 1 in intestinal mucosa lamina propria, monocytes and vascular endothelial cells is very low, and the affinity of its ligand LFA-1 also decreases accordingly (37). Consistent with previous reports in IBD patients (32) and DSS treated mice (38), we found that the protein expression of ICAM-1 was significantly increased in the model group. This result was further confirmed by Western blotting and immunohistochemical analysis. In view of the fact that TNF-α, IL-1β and IL-6 could actively up-regulate ICAM-1 expression and that increased serum levels of these cytokines were detected in DSS model, the increased expression of ICAM-1 might be due to the increased cytokines. Thus, the inhibitory effect of neferine may result in its effect on cytokines secretion. However, the detailed effect and mechanism of ICAM- 1 need further investigation. Collectively, the protective effect of neferine was summarized as Figure 6.

## Conclusion

According to our results, intraperitoneal injection of neferine had protective effect on UC in a DSS-induced experimental mice model; this effect was mediated through regulating inflammatory responses and COX-2, iNOS, and ICAM-1 expressions.
